# Review of headache in pregnancy

**Published:** 2012-11

**Authors:** Nasrin Jalilian, Taravat Fakheri, Sara Daeichin

**Affiliations:** ^*a*^Maternity Research Center, Kermanshah University of Medical Sciences, Kermanshah, Iran.

## Abstract

**Background:**

Headache is one of the most frequent reasons for referral to a neurology department. Headaches may occur at any time during pregnancy, but they tend to be most common during the first and third trimesters.The aim of this study is to review of headache in pregnancy.

**Etiology:**

An increase in headaches during the first trimester is believed to be caused by the surge of hormones along with an increase in the blood volume. These headaches may be further aggravated by stress, poor posture or changes in vision. Other causes of headaches during pregnancy may involve by Lack of sleep ,Low blood sugar , Dehydration ,Caffeine withdrawal ,Stress .Women who have regular migraine headaches may discover that they experience fewer migraines during pregnancy; however, some women may encounter the same number or even more migraine headaches. Headaches during the third trimester tend to be related more to poor posture and tension from carrying extra weight. Headaches during the third trimester may also be caused by preeclampsia.

**Treatment:**

Drugs are commonly used during pregnancy despite insufficient knowledge about their effects on the growing fetus. Most drugs are not teratogenic but Nonpharmacologic treatment is the ideal solution; however, analgesics such as acetaminophen and opioids can be used on a limited basis.

**Conclusions:**

Most headaches follow a benign course during pregnancy, although migraine is associated with increased risk of hypertensive disorders of pregnancy and stroke. Management of primary headaches during pregnancy is essentially similar to management in the nonpregnant state, with a few exceptions.

**Keywords:**

Headache, Pregnancy, Review


					Volume 4, Suppl. 1 Nov 2012
Publisher: Kermanshah University of Medical Sciences
URL: http://www.jivresearch.org
Copyright:
Congress of Iranian Neurosurgeons, 14-16 November 2012, Kermanshah, Iran
Paper No. 79
Review of headache in pregnancy
Nasrin Jalilian a
, Taravat Fakheri a,*
, Sara Daeichin a
a
Maternity Research Center, Kermanshah University of Medical Sciences, Kermanshah, Iran.
Abstract:
Background: Headache is one of the most frequent reasons for referral to a neurology department. Headaches may
occur at any time during pregnancy, but they tend to be most common during the first and third trimesters.The aim of
this study is to review of headache in pregnancy.
Etiology: An increase in headaches during the first trimester is believed to be caused by the surge of hormones along
with an increase in the blood volume. These headaches may be further aggravated by stress, poor posture or
changes in vision. Other causes of headaches during pregnancy may involve by Lack of sleep ,Low blood sugar ,
Dehydration ,Caffeine withdrawal ,Stress .Women who have regular migraine headaches may discover that they
experience fewer migraines during pregnancy; however, some women may encounter the same number or even more
migraine headaches. Headaches during the third trimester tend to be related more to poor posture and tension from
carrying extra weight. Headaches during the third trimester may also be caused by preeclampsia.
Treatment: Drugs are commonly used during pregnancy despite insufficient knowledge about their effects on the
growing fetus. Most drugs are not teratogenic but Nonpharmacologic treatment is the ideal solution; however,
analgesics such as acetaminophen and opioids can be used on a limited basis.
Conclusions: Most headaches follow a benign course during pregnancy, although migraine is associated with
increased risk of hypertensive disorders of pregnancy and stroke. Management of primary headaches during
pregnancy is essentially similar to management in the nonpregnant state, with a few exceptions.
Keywords:
Headache, Pregnancy, Review
* Corresponding Author at:
Taravat Fakheriemail: Associated professor of Obs & Gyn Department, Maternity Research Center of Kermanshah University of Medical Sciences,
Kermanshah, Iran, Email: tfakheri@kums.ac.ir, (Fakheri T.).

				

